# Clinical characteristics and outcomes of liver transplant recipients with carbapenem resistant klebsiella pneumonia infection

**DOI:** 10.3389/fcimb.2026.1864183

**Published:** 2026-07-23

**Authors:** Yan Dang, Ning Liu, Gengxia Yang, Ming Chen, Xiurong Ding, Fangfang Dai, Yanhua Yu, Jinli Lou

**Affiliations:** 1 Department of Clinical Laboratory Center, Beijing Youan Hospital, Capital Medical University, Beijing, China; 2 Department of General Surgery, Liver Transplantation Center, Beijing Youan Hospital, Capital Medical University, Beijing, China

**Keywords:** carbapenems, ceftazidime-avibactam, klebsiella pneumoniae, liver transplantation, outcomes

## Abstract

**Background:**

Carbapenem resistant klebsiella pneumoniae infection increased the mortality rate of liver transplant recipients. This study aims to provide more evidence for the clinical diagnosis and treatment of carbapenem resistant klebsiella pneumonia infection in liver transplant recipients.

**Methods:**

This study included liver transplant recipients who developed CRKP infections during the perioperative period from January 2019 to December 2024 at Beijing You’an Hospital. Statistical analysis was performed on their clinical and outcomes characteristics.

**Results:**

Among the 48 liver transplant recipients with CRKP infections, the 30 days mortality rate and the 90 days mortality rate after infection was 25%(12/48) and 31.25%(15/48). The mortality rates of patients with abdominal cavity infections were significantly higher than those of other patients (*p* < 0.05). The mortality rate of patients with mechanical ventilation and with septic shock during infection was higher (*p* < 0.05). Among the initial laboratory indicators after patient infection, there were significant differences in platelet (PLT), procalcitonin (PCT), total bilirubin (TBIL), creatinine (CR), and urea (UR) between the survival group and the death group (*p* < 0.05). Intra−abdominal infection (OR = 7.413, 95% CI: 1.218-45.136, *p* = 0.030) and septic shock (OR = 25.015, 95% CI: 4.128-151.594, *p* < 0.001) were both independent risk factors for 90−days mortality. The drug sensitivity results of CRKP strains from liver transplant recipients showed that the three drugs with the lowest resistance rates were ceftazidime-avibactam, polymyxin, and tigecycline. Different drugs and drug combinations did not cause differences in the all-cause mortality rates of patients after infection (*p* > 0.05). The risk of death in patients who received effective antibiotics within 48 hours was reduced by 54.3% (HR = 0.457, 95% CI: 0.163-1.285, *p* = 0.135), but due to the limited sample size, this result did not reach statistical significance.

**Conclusion:**

Clinicians should remain vigilant for abdominal infections, promptly control the infection source, and recognize early signs of septic shock to initiate timely treatment—key measures to reduce mortality for liver transplant recipients with CRKP infection. Additionally, effective antibiotic therapy within 48 hours after sampling may confer a survival benefit.

## Introduction

Liver transplantation (LT) is the only radical treatment for various end-stage liver diseases (Fishman, 2007). Most liver transplant recipients have experienced liver failure before surgery and may be accompanied by multiple organ dysfunctions such as those of the lungs and kidneys. The surgery takes a long time and causes significant trauma. After the operation, patients need to rely on ventilators and intensive care unit (ICU) treatment. Meanwhile, a large amount of immunosuppressants are used, which suppresses the immune system, making them a high-risk group for infections. Postoperative infection is an important factor affecting the prognosis of liver transplant patients. Among the pathogens causing infections after liver transplantation, bacterial infections predominate (Romero and Razonable, 2011; Kim, 2014; Phichaphop et al., 2020). Gram-negative bacteria are more commonly found among the pathogenic bacteria, and the mortality rate of recipients infected with Gram-negative bacteria is higher than that of those infected with Gram-positive bacteria (Chen et al., 2020; Phichaphop et al., 2020). Among the recipients infected with Gram-negative bacteria, Klebsiella pneumoniae is the most common (Schultze et al., 2021).

Klebsiella pneumoniae is an opportunistic nosocomial pathogen and an important pathogenic factor for nosocomial infections globally at present. It can cause various infectious diseases in adults and neonates, including pneumonia, urinary tract infections, abdominal infections, and sepsis. Once infected, there will be a relatively high morbidity and mortality rate (Aslan et al., 2022; Brescini et al., 2025; Zheng et al., 2025). In recent years, with the widespread use of carbapenem antibiotics, the risk of multi-drug resistant(MDR) bacteria infections, especially carbapenem resistant Klebsiella pneumoniae (CRKP) infections, in liver transplant recipients has been further exacerbated. Multiple studies have shown that the infection rate of CRKP is increasing year by year, which not only leads to a decrease in the survival rate of transplanted organs but also reduces the 3-month survival rate of liver transplant patients after surgery (Barchiesi et al., 2016; Bias et al., 2018; Liu et al., 2022; Liu et al., 2025). Multidrug resistance also poses a huge challenge to the selection of clinical antibiotics and treatment methods.

Previous research by our team (Liu et al., 2022) showed that in liver transplant recipients with CRKP infection, the length of ICU stay, pre-operation infection, and hepatocellular carcinoma were independent risk factors for CRKP infection in liver transplant patients. With COX multivariate regression analysis, the 3-months survival rate of CRKP infected patients was significantly lower than that without CRKP infection post-LT. In order to further analyze the clinical characteristics and prognosis of liver transplant recipients infected with CRKP, we conducted this study, aiming to provide more evidence for the clinical diagnosis and treatment of liver transplant recipients with CRKP infection.

## Materials and methods

### Research design and subjects

This study included 875 patients who underwent liver transplantation at Beijing You’an Hospital between January 2019 and December 2024. The inclusion criteria for the study were as follows: (1) Recipients of allogeneic liver transplantation; (2) Aged over 18 years; (3) Those who developed infections with carbapenem-resistant Klebsiella pneumoniae during the perioperative period after surgery. The exclusion criteria were: (1) Those aged under 18 years; (2) Those with incomplete clinical data. If a patient developed a second CRKP infection at a different site during the perioperative period, the patient was counted once. All the patients included were first-time transplant recipients, and there were no patients who underwent re-transplantation.

**Figure 1:**
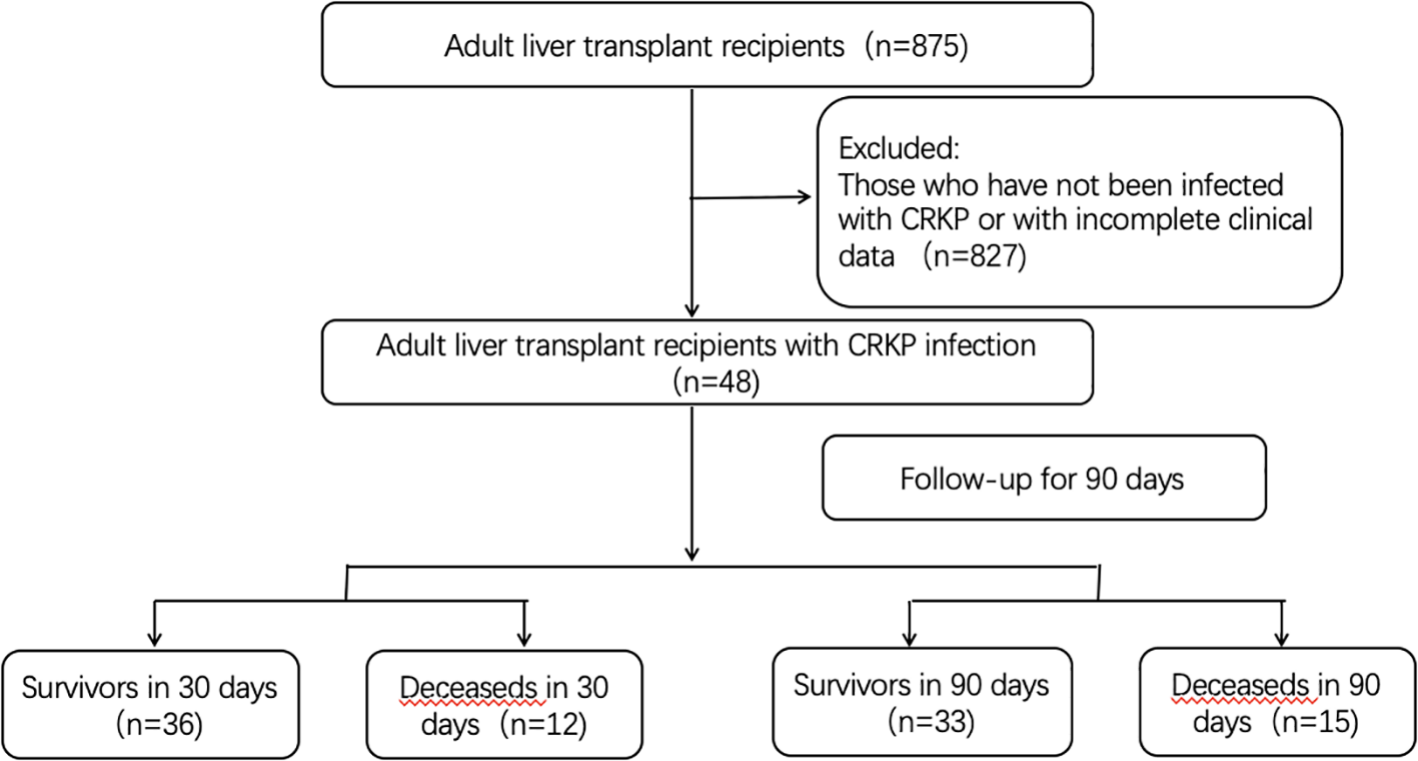
Patient flow diagram of this study.

All patients who received liver transplants were regularly followed up after the operation. None of the patients were found to be infected with CRKP in our center before the transplantation procedure. The follow-up endpoint of this study was 90 days after infection (**Figure 1**). All recruited transplant patients received immunosuppressive therapy. The intraoperative prophylactic medication and postoperative anti-infection treatment regimens were third generation cephalosporins or carbapenems. This study was approved by our institutional Ethics Review Committee (Jingyou kelunzi [2023] No. 089) and was conducted according to the Declaration of Helsinki principles.

### Clinical data collection

The following data were collected by accessing the hospital’s electronic medical record system, laboratory information system, and transplant specialty database:

1. Baseline data: Demographic characteristics (age and gender), primary diseases (hepatitis B cirrhosis, liver cancer, etc.), Model for End-stage Liver Disease (MELD) score, Child-Pugh classification, preoperative comorbidities (diabetes, hypertension, etc.), and preoperative diagnosis.
2. Intraoperative data: Surgical approach, operation duration, intraoperative blood loss, transfusion volume, and the results of organ preservation solution culture.
3. Data related to postoperative infection: Time of infection occurrence, site of infection, immunosuppressive regimen at the time of infection, infection complications, initial laboratory indicators after infection, targeted anti-infection treatment regimen after infection.
4. Outcome indicators: The survival status of patients 30 days after infection, and patients are divided into the 30-days survival group and the 30-days death group accordingly; The survival status of patients 90 days after infection, and patients are divided into the 90-days survival group and the 90-days death group accordingly.

### Microbiological analysis

During the follow-up period, once infection is suspected, clinical samples are collected for culture. Strains are identified using the automatic rapid biomass mass spectrometry detection system (BRUKER, Germany), and the test for susceptibility to antibiotics is carried out using the Phoenix 100 automatic system (BD, USA). The susceptibility test of ceftazidime-avibactam was conducted using the E-Test method. The results were interpreted using the Clinical and Laboratory Standards Institute (CLSI) breakpoints (Humphries et al., 2021).

### Definitions

1. Perioperative period was defined as the continuous time from the patient’s admission for surgical treatment to the discharge after the completion of all treatments related to the current surgery.
2. CRKP was defined as insensitivity to at least one carbapenems, with a minimum inhibitory concentration ≥2 ug/mL for ertapenem or ≥4 ug/mL for imipenem or meropenem.
3. Carbapenem resistant Klebsiella pneumoniae infection was defined as that on the basis of meeting the clinical diagnostic criteria for the corresponding infection site (Horan et al., 2008) (such as fever, local inflammatory signs, abnormal imaging findings), CRKP is isolated from a sterile site (such as blood, peritoneal fluid) or a qualified nonsterile site specimen (such as sputum).
4. Effective antibiotic treatment was defined as the use of drugs that are sensitive according to *in vitro* susceptibility test results for antibacterial treatment.

### Statistical analysis

Statistical analysis was performed using SPSS 26.0 (IBM, USA). Measurement data conforming to the normal distribution were expressed as Mean ± Standard deviation, and the independent- samples t-test was used for comparison between groups; data with non-normal distribution were expressed as median (interquartile range), and the Mann-Whitney U test was employed. Count data were expressed as frequency (percentage), and the Fisher’s exact test was used for comparison between groups. The Bonferroni method was used to adjust for multiple comparisons of rates among multiple groups. The risk factors for 90-days mortality in LT recipients with CRKP infection were identified by multivariate logistic regression analyses using odds ratios (ORs) and 95% confidence intervals (CIs). Kaplan-Meier curves with log-rank test and univariate Cox regression (HR, 95% CI) were performed to explore the effective antibiotics within 48 hours’ effect on survival time. P < 0.05 was considered statistically significant.

## Results

### Study population and clinical baseline characteristics

Among 875 liver transplant patients, 48 patients developed CRKP infection after the operation, with an infection rate of 5.49%. Among the 48 patients, 12 died within 30 days, with a mortality rate of 25%, and 15 died within 90 days, with a mortality rate of 31.25%. There were 29 males (60.42%, 29/48) and 19 females (39.58%, 19/48), and the median age was 55 years old. Gender and age did not cause differences in the mortality rate of patients. Before the operation, 23 patients (47.92%, 23/48) had anemia, 10 patients (20.83%, 10/48) had hypertension, 8 patients (16.67%, 8/48) had diabetes, and 5 patients (10.42%, 5/48) had heart disease. There was a marginal difference in the 90-days survival and 90 -days death groups among patients with hypertension (*p* = 0.051), and other comorbidities did not cause differences in the mortality rate of patients. Before the operation, 34 patients (70.83%, 34/48) were diagnosed with liver failure, and 14 patients (29.17%, 14/48) were diagnosed with primary liver cancer. The most common etiologies of the patients were hepatitis B virus and alcoholic hepatitis, with 12 cases (25.00%, 12/48) and 11 cases (22.92%, 11/48) respectively. Different diagnoses and etiologies of the patients did not cause differences in the mortality rate. The preoperative Model for End-Stage Liver Disease score(MELD) score, Child-Turcotte-Pugh(CHILD) classification of the patients and pre-transplant renal function also had no significant impact on the mortality rate (**Table 1**).

**Table 1:** Clinical baseline characteristics of liver transplant recipients with CRKP, n(%) .

Variables	All (n=48)		Survival in 30 days (n=36)	Death in 30 days (n=12)		*P*	Survival in 90 days (n=33)	Death in 90 days (n=15)	*P*
Sex						1.000			1.000
Male	29 (60.42%)		22	7			20	9	
Female	19(39.58%)		14	5			13	6	
Age(years)	55(43.25,58)		50(38.75,57)	55(51.25,61.75)		0.076	49(39.5,57.5)	55(51,58)	0.164
Preoperative comorbidities									
Anemia	23(47.92%)	19		4	0.324		18	5	0.221
Hypertension	10(20.83%)	5		5	0.094		4	6	0.051
Diabetes	8(16.67%)	7		1	0.659		6	2	0.714
Heart Disease	5(10.42%)	2		3	0.092		2	3	0.092
Preoperative diagnosis					0.294				0.094
Hepatic failure	34(70.83%)	27		7			26	8	
Hepatocellular carcinoma	14(29.17%)	9		5			7	7	
Pathogenesis					0.594				0.689
Hepatitis B	12(25.00)	9		3			8	4	
Alcoholic liver disease	11(22.92%)	8		3			7	4	
Medicamentous liver lesion	6(12.50%)	5		1			4	2	
Autoimmune hepatitis	3(6.25%)	2		1			2	1	
Biliary tract obstruction	2(4.17%)	1		1			1	1	
Hepatolenticular degeneration	2(4.17%)	1		1			1	1	
Hepatitis C	1(2.08%)	0		1			0	1	
Multi-factor	3(6.25%)	3		0			3	0	
Unknow	8(16.67%)	7		1			7	1	
MELD score	23.17 ± 8.74	23.14 ± 8.63		23.25 ± 9.46	0.972		23.48 ± 8.72	22.47 ± 9.06	0.718
Child-Pugh Classification					0.081				0.153
A	3(6.25%)	1		2			1	2	
B	11(22.92%)	7		4			6	5	
C	34(70.83%)	28		6			26	8	
Pre-transplant renal function									
CR(umol/L)	64(48.25,107)	60.5(46.25,103)		73(65.25,126.75)	0.116		59(46.5,102)	72(64,110)	0.197
UR(mmol/L)	6.92(4.36,13.34))	6.55(3.96,11.81)		9.16(5.51,19.64)	0.078		6.44(4.01,11.08)	7.90(5.24,13.68)	0.151

CR, creatinine; UR, urea: Data with the normal distribution were expressed as Mean ± Standard; Data with non-normal distribution were expressed as median (interquartile range).

Before the detection of CRKP, 44 out of 48 recipients (91.67%) had a history of exposure to carbapenem drugs. Among them, 38 cases (79.17%) had a history of carbapenem drug exposure one week before the detection of CRKP, and 6 cases (12.5%) had a history of carbapenem drug exposure two weeks before the detection of CRKP.

### Intraoperative conditions of liver transplant recipients infected with CRKP

42 cases (87.50%, 42/48) underwent classic orthotopic liver transplantation, 5 cases (10.42%, 5/48) underwent piggyback liver transplantation, and 1 case (2.08%, 1/48) underwent split liver transplantation. Different surgical methods did not have a significant impact on the mortality rate of patients. The surgical duration, blood loss, and red blood cell transfusion volume of patients also did not cause differences in the mortality rate (**Table 2**). CRKP was detected in the organ preservation solution of 12 cases (25.00%, 12/48), but it did not cause differences in the mortality rate of patients.

**Table 2:** Intraoperative conditions of liver transplant recipients with CRKP, n(%).

Variables	All (n=48)	Survival in 30 days(n=36)	Death in 30 days (n=12)	*P*	Survival in 90 days(n=33)	Death in 90 days (n=15)	*P*
Surgical method				1.000			1.000
Standard Orthotopic Liver Transplantation	42(87.50%)	31	11		28	14	
Piggyback liver transplantation	5(10.42%)	4	1		4	1	
Split liver transplantation	1(2.08%)	1	0		1	0	
Duration of the surgery(min)	405(361.5,490)	405(371.75,462)	415(350.25,497.50)	0.981	390(365,480.5)	429(351,490)	0.722
Blood loss volume(ml)	1100(600,1700)	1150(600,1675)	950(625,3375)	0.971	1100(600,1650)	1000(600,3000)	0.876
Red blood cell infusion volume(ml)	1200(800,2000)	1200(800,1900)	1200(800,2825)	0.990	1200(800,1800)	1200(800,2000)	0.830
CRKP detected in the organ preservation solution				1.000			1.000
Yes	12(25.00%)	9	3		8	4	
No	36(75.00%)	27	9		25	11	

Data with non-normal distribution were expressed as median (interquartile range).

### The patient’s condition during the period of CRKP infection after the operation

Among patients with CRKP infection after liver transplantation, the most common infection site was the respiratory tract (64.58%, 31/48), followed by the abdominal cavity (43.75%, 21/48) and peripheral blood (22.92%, 11/48). The 30-days and 90-days mortality rates of patients with abdominal cavity infection after infection were significantly higher than those of patients with infections in other sites (*p* = 0.018 and 0.011). 30 (62.50%, 30/48) had single-site infections, and 18 (37.50%, 18/48) had multi-site infections. There was a statistically significant difference in 30−day mortality among patients with different numbers of infected sites, whereas the difference in 90−day mortality did not reach statistical significance (*p* = 0.002 and 0.007) (**Table 3**).

**Table 3:** The characteristics of infection sites in liver transplant recipients with CRKP, n(%).

Variables	All (n=48)	Survival in 30 days(n=36)	Death in 30 days (n=12)	*P*	Survival in 90 days(n=33)	Death in 90 days (n=15)	*P*
Infection sites							
Respiratory tract	31(64.58%)	23	8	1.000	21	10	1.000
Abdominal cavity	21(43.75%)	12	9	**0.018**	10	11	0.011
Peripheral blood	11(22.92%)	7	4	0.430	6	5	0.283
Biliary tract	7(14.58%)	5	2	1.000	5	2	1.000
Others	8(16.67%)	6	2	1.000	3	1	1.000
Number of infected sites				0.002*			0.007*
1	30(62.50%)	27	3		25	5	
2	9(18.75%)	5	4		5	4	
3	7(14.58%)	2	5		2	5	
4	1(2.08%)	1	0		0	1	
5	1(2.08%)	1	0		1	0	

*P < 0.005 was considered statistically significant after correction. Bold values indicated that these variables were significant.

The median time from surgery to the first detection of CRKP was 6.5 days and The longest duration is 35 days. The immunosuppressants commonly used were tacrolimus, mycophenolic acid, and glucocorticoids. There wew no difference in the immunosuppressive regimens between patients who survived and died. Among the 48 patients, 33 (68.75%) were detected with CRKP infection in the ICU ward, and 15 (31.25%) were detected with CRKP infection in the general surgical ward. The 30-days and 90-days mortality rates of patients detected with CRKP infection in the ICU ward were higher than those of patients in the general surgical ward (*p* = 0.009 and 0.002) (**Table 4**).

**Table 4:** Postoperative infection characteristics of liver transplant recipients with CRKP, n(%).

Variables	All (n=48)	Survival in 30 days(n=36)	Death in 30 days (n=12)	*P*	Survival in 90 days(n=33)	Death in 90 days (n=15)	*P*
The duration from the surgery to the first detection(days)	6.5(2,13)	6(2,12.75)	8.5(2.25,19.5)	0.452	7(2.5,12.5)	6(1,21)	0.713
Immunosuppressant							
Tacrolimus	41(85.41%)	33	8	0.055	32	9	0.002
Mycophenolic acid	36(75.00)	28	8	0.462	26	10	0.476
Glucocorticoids	34(70.83%)	26	8	0.726	24	10	0.738
Basiliximab	5(10.42%)	2	3	0.092	2	3	0.307
Cyclosporin	2(4.17%)	1	1	0.441	0	2	0.093
Department during infection				0.009			0.002
ICU	33(68.75%)	21	12		18	15	
Surgical ward	15(31.25%)	15	0		15	0	
Condition during infection							
mechanical ventilation	26(54.17%)	16	10	**0.023**	14	12	**0.027**
Continuous renal replacement therapy	12(25.00%)	8	4	0.462	7	5	0.476
Respiratory failure	11(22.91%)	7	4	0.430	6	5	0.283
Septic shock	17(35.42%)	7	10	**<0.001**	5	12	**<0.001**
Infections by other pathogens	35(72.92%)	25	10	0.469	22	13	0.182
Second operation	6(12.5%)	4	2	0.631	3	3	0.360
Acute rejection	11(22.91%)	10	1	0.248	9	2	0.462

Data with the normal distribution were expressed as Mean ± Standard; Data with non-normal distribution were expressed as median (interquartile range). Bold values indicated that these variables were significant.

The situations of mechanical ventilation, continuous renal replacement therapy (CRRT), respiratory failure, septic shock, co-infection with other pathogens, secondary surgery, and acute rejection during the patients’ infection period were analyzed. The results showed that the 30-days and 90-days mortality rates of patients with mechanical ventilation and septic shock during infection were higher (*p* = 0.023 and 0.027, *p* < 0.001), and there were no significant differences in the 30-days and 90-days mortality rates of patients with other concurrent conditions (**Table 4**).

**Figure 2:**
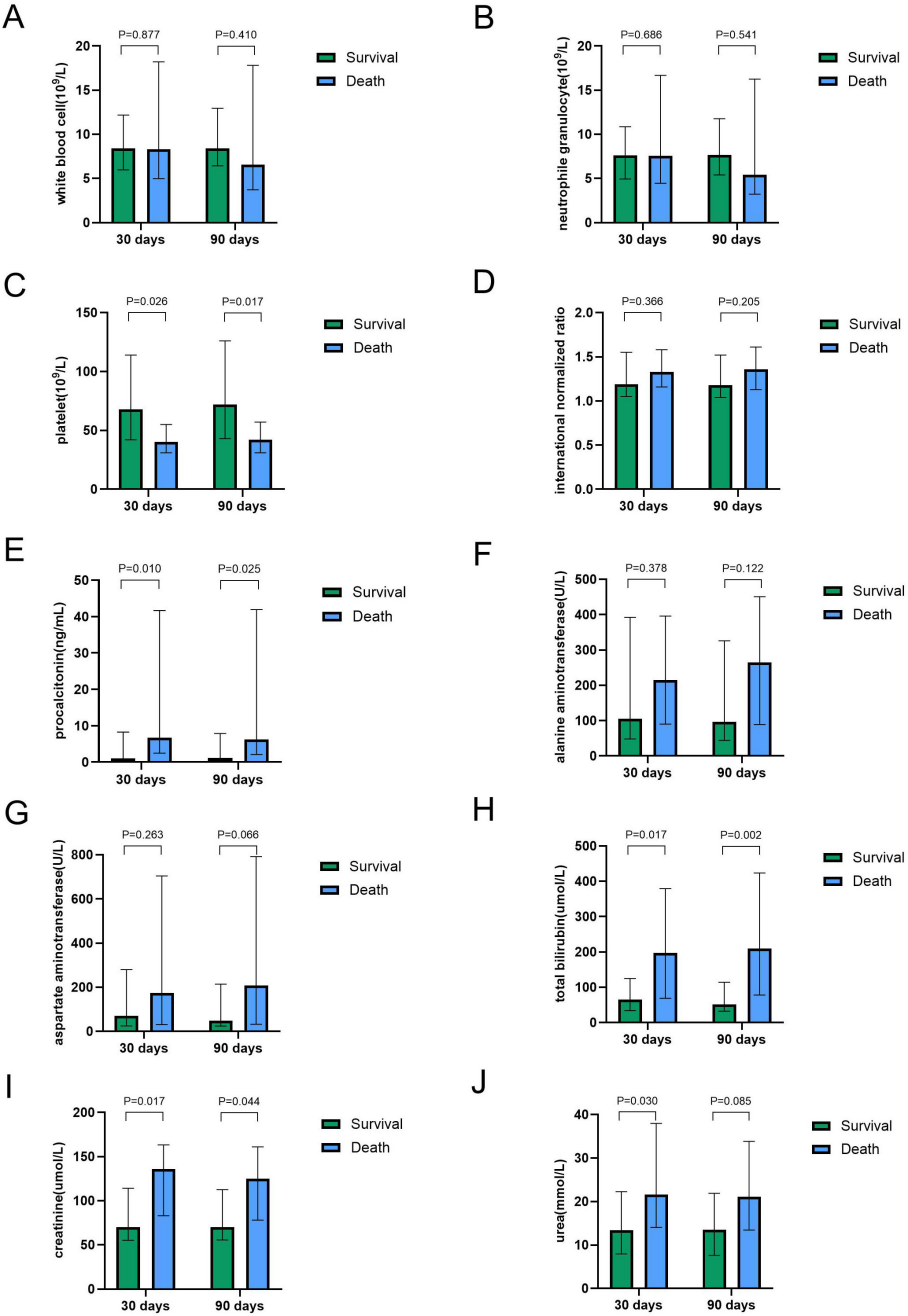
Comparison of initial laboratory indicators between the death group and the survival group after infection.

The initial laboratory indicators after patients’ infection were analyzed. The results showed that there were no significant differences in white blood cell (WBC) count, neutrophile granulocyte (NET) count, international normalized ratio (INR), alanine aminotransferase (ALT) and aspartate aminotransferase (AST) between the survival group and the death group at 30-days and 90-days. There were significant differences in platelet (PLT), procalcitonin (PCT), total bilirubin (TBIL), creatinine (CR), and urea (UR) levels between the survival group and the death group at 30-days and 90-days (*p* = 0.026 and 0.017, *p* = 0.010 and 0.025, *p* = 0.017 and 0.002, *p* = 0.017 and 0.044, *p* = 0.030 and 0.085) (**Figure 2**).

### Risk factors for 90−days mortality in LT recipients with CRKP infection

Among liver transplant recipients with carbapenem−resistant Klebsiella pneumoniae (CRKP) infection, given the small sample size, we selected the two clinical indicators with the greatest statistical effect sizes, intra−abdominal infection and septic shock for inclusion in a multivariate logistic regression analysis to assess their association with 90−day mortality. The results showed that intra−abdominal infection (OR = 7.413, 95% CI: 1.218-45.136, *p* = 0.030) and septic shock (OR = 25.015, 95% CI: 4.128-151.594, *p* < 0.001) were both independent risk factors for 90−day mortality, with statistical significance (**Table 5**).

**Table 5:** Multivariate analysis of the risk of 90-days mortality in LT recipients with CRKP infection.

Variable	Death in 90 days		Multivariate analysis	
	Yes (n=15)	No (n=33)	OR (95%CI)	*P*
intra−abdominal infection(n,%)	11(73.33%)	10(30.30%)	7.413(1.218,45.136)	**0.03**
Septic shock(n,%)	12(80.00%)	5(15.15%)	25.015(4.128,151.594)	**<0.001**

Bold values indicated that these variables were significant.

### Antibiotics sensitivity of strains

**Figure 3:**
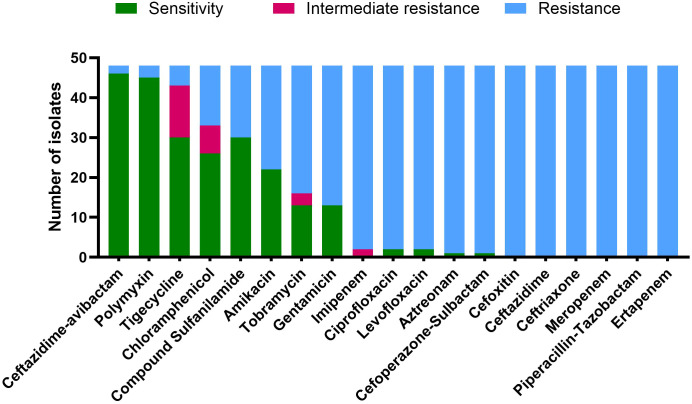
The results of the antibiotics sensitivity test for carbapenem-resistant klebsiella pneumonia.

All strains were resistant to cefoxitin, ceftazidime, ceftriaxone, piperacillin-tazobactam, meropenem, and ertapenem (**Figure 3**). The resistance rates to imipenem, ciprofloxacin, levofloxacin, aztreonam, and cefoperazone-sulbactam were all above 95%. The three drugs with the lowest resistance rates were ceftazidime-avibactam, polymyxin, and tigecycline, with resistance rates of 4.17% (2/48), 6.25% (3/48), and 10.42% (5/48) respectively.

### Comparison of different antibiotic regimens and survival analysis of effective antibiotics within 48 hours

42 cases (87.50%, 42/48) were treated with polymyxin, tigecycline, and ceftazidime-avibactam as antibacterial agents. All-cause mortality of patients treated with ceftazidime-avibactam monotherapy or combination was not superior to that of patients treated with other antibiotics, and the difference was not significant (*p* = 0.739 and 0.341). Different antibiotic treatment regimens, including single drug therapy with various medications and combined therapy with multiple drugs, also did not result in differences in the all-cause mortality rate of the patients. (*p* = 0.516 and 0.766) (**Table 6**).

**Table 6:** Analysis of the clinical application of antibiotics of liver transplant recipients with CRKP, n(%).

Variables	All (n=48)	Survival in 30 days(n=36)	Death in 30 days (n=12)	P	Survival in 90 days(n=33)	Death in 90 days (n=15)	P
Use of effective antibiotics within 48 hours after sampling	27(56.25%)	22	5	0.200	21	6	0.112
use of Ceftazidime-avibactam	29(60.42%)	21	8	0.739	18	11	0.341
Antibacterial therapeutic drugs				0.516			0.766
Ceftazidime-avibactam	3(6.25%)	1	2		1	2	
Tigecycline	6(12.50%)	5	1		5	1	
Polymyxin	3(6.25%)	2	1		2	1	
Ceftazidime-avibactam and Tigecycline	10(20.83%)	9	1		6	4	
Tigecycline and Polymyxin	4(8.33%)	4	0		4	0	
Ceftazidime-avibactam and Polymyxin	4(8.33%)	3	1		3	1	
Ceftazidime-avibactam,Tigecycline and Polymyxin	12(25.00%)	8	4		8	4	
Carbapenems	6(12.50%)	4	2		4	2	

The median time for the patient to start receiving effective antibiotic treatment was 2 days. At present, the conventional culture, identification and antibiotics sensitivity testing methods usually take about at least two days to confirm CRKP infection. A total of 27 cases (56.25%, 27/48) received effective antibiotics within 48 hours after sampling. Their 30-days and 90-days all-cause mortality were 18.82% (5/27) and 22.22% (6/27) respectively, which were lower than that of cases not received effective antibiotics within 48 hours (33.33%, 7/21 *vs* 42.86%, 9/21), but the differences were not significant (*p* = 0.200 and 0.112).

**Figure 4:**
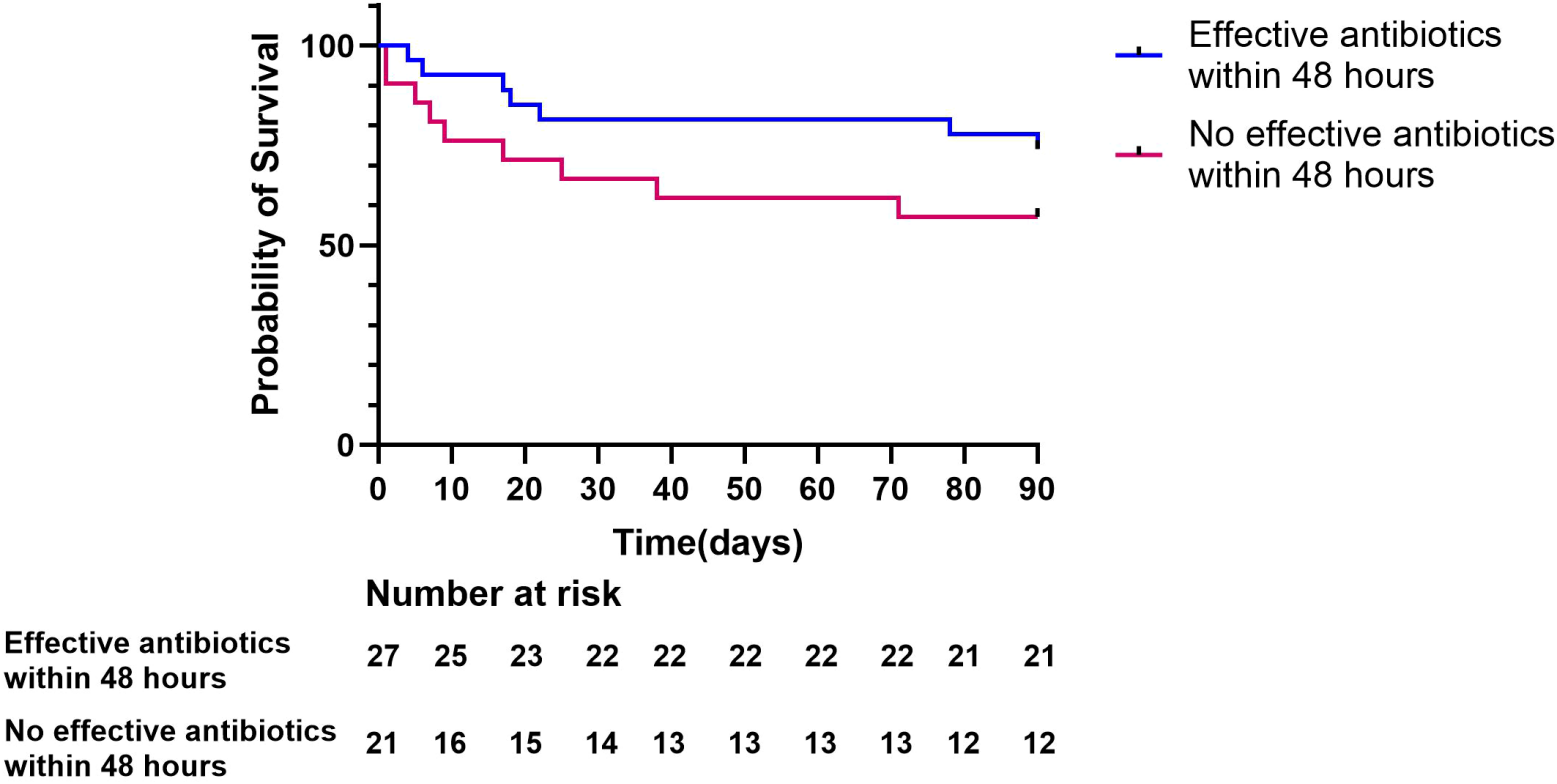
Kaplan-Meier curves for all-cause mortality: Survival for LT with CRKP infection within 90 days by effective antibiotics within 48 h.

The Kaplan-Meier survival curve showed that during the entire follow-up period, the survival curve of patients who received effective antibiotics within 48 hours after sampling was always above that of patients who did not receive effective antibiotics within 48 hours after sampling, presenting a continuous separation trend (**Figure 4**). However, limited by the small sample size, the Log-rank test showed that the difference in overall survival between the two groups did not reach the statistical significance level (*p* = 0.127). Univariate Cox regression analysis showed that the risk of death in patients who received effective antibiotics within 48 hours after sampling was reduced by 54.3% (HR = 0.457), but this result did not reach statistical significance (95% CI: 0.163-1.285, *p* = 0.135).

## Discussion

In our study, the 30-days and 90-days mortality rates of liver transplant recipients infected with CRKP were 25% and 31.25% respectively, which were lower than the previous reports (Lu et al., 2023). The basic conditions such as the patients’ age, gender, other comorbidities, diagnosis, and etiology did not show differences between the death group and the survival group. The intraoperative conditions of patients, including the surgical procedure, operation duration, blood loss, and the amount of red blood cell transfusion, did not have a significant impact on the mortality rate of patients after infection. CRKP was detected in the organ preservation solution of 25.00% of liver transplant recipients. However, the infections possibly from the donors might not have caused a difference in the mortality rate of the patients.

Tao-Hua Liu (Liu et al., 2025) found that the lungs and blood were the most common sites of CRKP infection in liver transplant recipients, this study shows that the most common infection site is the respiratory tract, followed by the abdominal cavity and peripheral blood, which is basically consistent with their results. Meanwhile, the mortality rates of recipients with intra−abdominal infection were significantly higher than other LT recipients in our study.

This study shows that different types of immunosuppressant, CRRT, respiratory failure, co-infection with other pathogens, secondary surgery, and acute rejection during infection did not affect the patient’s mortality rate. However, the mortality rate of ICU-infected patients was higher than that of patients infected in the general surgical ward. The mortality rate of patients with mechanical ventilation and septic shock during infection was even higher. In particular, for patients with septic shock, the difference in mortality rate was extremely significant.

This study demonstrates that intra−abdominal infection and septic shock are significant independent predictors of 90−day mortality in liver transplant recipients with CRKP infection. Although the number of cases was limited, both indicators exhibited strong effect sizes, highlighting their importance in prognostic assessment. Intra−abdominal infection can trigger local and systemic inflammatory responses, disrupt intestinal barrier function, promote bacterial translocation, and thereby exacerbate the severity of CRKP infection (Assimakopoulos et al., 2024). In liver transplant recipients, uncontrolled intra−abdominal infection can rapidly progress to sepsis or even multiple organ failure. Septic shock, as the terminal stage of severe sepsis, once developed, often indicates an irreversible condition with a sharp increase in mortality. Similar to our research results, previous studies (Liu et al., 2023; Lu et al., 2023) also showed that septic shock is an independent risk factor for the death of organ transplant recipients after CRKP infection.

Among the initial laboratory indicators after the patient’s infection, there were significant differences in the levels of PLT, PCT, TBIL, CR, and UR between the survival group and the death group. This suggests that higher bilirubin levels, infection indicators, and poor renal function are all associated with a poor prognosis for patients. PLT not only participate in physiological hemostasis but also play a major role in liver ischemia-reperfusion injury, liver damage, tissue repair, and liver regeneration. A decrease in platelet count can lead to spontaneous bleeding, weaken the defense mechanism against infections, and other complications that can seriously impact patient prognosis (Ma et al., 2024). PCT, as a sensitive inflammatory marker, its elevation precisely alerts and marks the vicious cycle of uncontrolled infection, inflammatory storm, and immune disorder. This, in turn, indirectly leads to a high mortality rate among liver transplant recipients when they are infected with drug-resistant bacteria (Satija et al., 2024). The increase in total bilirubin leads to a direct impairment of the neutrophils’ oxygen-free radical killing function, triggers a systemic immunosuppressive state, and reversely warns of graft dysfunction. Together, these factors form a lethal synergy with drug-resistant bacterial infections, systematically amplifying the mortality risk for liver transplant recipient (Arai et al., 2001). Previous studies (Liu et al., 2023; Liu et al., 2025) also showed that a higher total bilirubin level is an independent risk factor for the death of transplant patients after infection and is more likely to cause sepsis in patients. For liver transplant recipients with elevated creatinine and urea levels, due to the immune paralysis caused by renal dysfunction and abnormal drug metabolism, they are more likely to die after being infected with drug-resistant bacteria. This is because they are prone to experiencing uncontrolled systemic inflammatory responses, limited antibacterial treatment, and the fatal cycle of “renal injury - liver injury - renal injury” (Gatti and Pea, 2021; Zhou et al., 2026). It is worth noting that patients with poor renal function should receive more clinical attention in liver transplant recipients with CRKP infection. More research is needed to confirm the predictive value of these laboratory indicators in terms of warning of death in liver transplant recipients with drug-resistant bacterial infections.

In this study, the antibiotics sensitivity results of CRKP strains from liver transplant recipients showed that the three drugs with the lowest resistance rates were polymyxin, tigecycline, and ceftazidime-avibactam. The results were basically consistent with the antibiotics resistance situations of strains in patients with CRKP infection after various organ transplants such as liver transplantation, kidney transplantation, and hematopoietic stem cell transplantation reported in previous literatures (Wang et al., 2021; Liu et al., 2022; Wu et al., 2022; Long et al., 2024). Meanwhile, ceftazidime-avibactam, polymyxin and tigecycline are also the most frequently used as antibiotics for treatment in our center.

Similar to our findings, the studies by Lu et al. (2023) and Elena Pérez-Nadales et al. (2023) indicated that the use of ceftazidime-avibactam did not reduce the mortality rate of patients. However, more studies (Chen et al., 2021; Zhang et al., 2021; Zheng et al., 2021; Zheng et al., 2022; Zheng et al., 2022; Hu et al., 2024; Zhang et al., 2024) have shown that the use of ceftazidime-avibactam can reduce the mortality rate of organ transplant patients after infection. Such inconsistent research results require more clinical data for judgment.

Research by Eleni Massa (Massa et al., 2025) shows that identifying MDR colonization and administering targeted perioperative antibiotics may reduce septic complications in liver transplant patients. Research by Jingli Lu (Lu et al., 2023) shows that when solid organ transplant recipients are infected with CRKP, receiving appropriate targeted treatment is associated with a reduced risk of death, but there is no difference in the mortality rate of patients treated with effective antibiotics within or without 24 hours. In our study, patients treated with effective antibiotics within 48 hours after sampling showed potential survival benefits (HR = 0.457), with a 54.3% reduction in the risk of death. Though, limited by the sample size, this result did not reach statistical significance. At present, the conventional culture, identification and antibiotics sensitivity testing methods usually take about at least two days to confirm CRKP infection. Improving the speed of laboratory testing, such as developing tests for drug-resistant genes, and early identification of antibiotics resistant bacterial infections are conducive to improving the clinical outcomes of patients.

The limitation of this study lies in the relatively small sample size. More multivariate analysis cannot be conducted and no significant difference in survival due to different treatment times could be identified. The research conclusions need to be verified in larger-scale, multi-center prospective studies. This study did not systematically collect information on pre-transplant immunosuppression, thus, its potential impact on post-transplant outcomes could not be analyzed. Meanwhile, more work is needed to carry out CRKP strains’ molecular or genotypic characterization and MDRO colonization. However, the current results provide valuable clues for future clinical research and practice.

In summary, for liver transplant recipients with CRKP infection, clinicians should remain vigilant for abdominal infections, promptly control the infection source, and recognize early signs of septic shock to initiate timely treatment—key measures to reduce mortality. Additionally, effective antibiotic therapy within 48 hours after sampling may confer a survival benefit.

## Data Availability

The original contributions presented in the study are included in the article/supplementary material. Further inquiries can be directed to the corresponding authors.
